# Extended platelet rich fibrin versus allograft as a space filling material in immediate dental implant placement

**DOI:** 10.1186/s12903-026-08891-6

**Published:** 2026-06-29

**Authors:** Hala Mohammed Mohammed Ahmed Elnaggar, Nesma El-Gohary, Ayman Ahmed Mustafa Yaseen, Mohamed Abdel-Moniem Tawfik

**Affiliations:** 1 Department of Oral and Maxillofacial Surgery, Faculty of Dentistry, Mansoura University, Mansoura, Egypt; 2 Department of Fixed Prosthodontics, Faculty of Dentistry, Mansoura University, Mansoura, Egypt

**Keywords:** Allograft, e-PRF, Implant stability, Immediate implant, Space filling, Gap filling

## Abstract

**Background:**

Immediate implant has been one of the solutions to decrease bone resorption after tooth extraction. Gap distance between the dental implant and adjacent bone has undergone much research to enhance bone formation in this area. This is small exploratory study that aimed to compare the efficacy of extended platelet rich fibrin (e-PRF) a regenerative biomaterial produced by heating platelet-poor plasma (PPP) to denature albumin, extending the resorption time of standard PRF from 2 to 3 weeks to 4–6 months, as well as allograft as a space filling material in immediate dental implant placement.

**Patients and Methods:**

Twenty-eight implants were placed for the prosthetic replacement of non-restorable single-rooted maxillary teeth using immediate implant placement. Patients were randomly allocated into two equal groups (*n* = 14 each). In Group I, allograft was placed in the peri-implant gap, whereas in Group II, e-PRF was used. Implant stability was assessed using resonance frequency analysis (RFA). Buccal bone thickness and marginal bone loss were evaluated using CBCT throughout the follow-up period. Statistical analysis was performed on all clinical and radiographic data.

**Results:**

A total of 28 patients received 28 immediate implants in the maxillary esthetic zone. During the 12-month follow-up period, all implants were successful with no complications. No statistically significant differences were observed between the groups regarding implant stability, marginal bone loss, or buccal bone thickness.

**Conclusion:**

Within this small exploratory trial, no statistically significant intergroup differences were detected between e-PRF and allograft as space-filling materials around immediate implants regarding stability, marginal bone loss, and buccal bone thickness.

**Trial registration:**

Clinical-Trials.gov PRS (https://register.clinicaltrials.gov) has this study registered under the identifier number. NCT07445776 on 03/02/2026.

**Supplementary Information:**

The online version contains supplementary material available at 10.1186/s12903-026-08891-6.

## Background

Immediate dental implants have transformed implant dentistry by significantly reducing treatment duration. The placement of implants immediately into fresh extraction sockets was first reported by Heimke and Schulte [1].

Immediate dental implant placement in freshly extracted sockets is now a well-established procedure. Previous studies have demonstrated that the success rate of implants placed immediately after tooth extraction is comparable to that of implants placed in healed sites [2, 3].

Due to its numerous advantages, including improved soft tissue esthetics, reduced treatment time, and fewer surgical interventions, which collectively decrease patient morbidity, immediate implant placement has become a widely accepted treatment protocol [4].

In addition, it contributes to the preservation of the soft tissue profile by maintaining the remaining alveolar bone. However, some authors have argued that immediate implant placement does not prevent the post-extraction bone resorption process [5].

Despite its advantages, immediate implant placement (IIP) presents several challenges, including difficulty in achieving the ideal implant position, challenges in obtaining primary stability, the inability to routinely assess all areas of the extraction site for infection, and difficulties in osteotomy preparation due to bur skiving along the extraction socket walls [6].

The morphology of extraction socket walls is often irregular, which may result in a considerable gap between the implant surface and the buccal alveolar bone. This represents one of the major differences between implants placed in fresh extraction sockets and those placed in healed sites. These defects have both vertical and horizontal components. Proper management of the horizontal component is particularly important for implant success. The horizontal gap distance, commonly referred to as the “jumping distance,” represents the distance that bone-forming cells must bridge between the implant surface and the surrounding bone [7].

Therefore, understanding the biological limitations associated with bridging this horizontal gap is essential. Appropriate management of this space is critical for achieving successful implant outcomes in terms of both function and esthetics [8].

Bone regeneration can occur spontaneously in gaps smaller than 2 mm without the need for barrier membranes or grafting materials. However, larger gaps may adversely affect the success of immediate implant placement [9, 10]. The optimal surgical approach for managing the buccal gap remains controversial. The primary objectives are to achieve adequate bone fill within the gap, maximize bone-to-implant contact (BIC), minimize buccal bone resorption, and reduce soft tissue recession [11].

A range of improved surgical techniques and augmentation strategies using barrier membranes and grafting materials, including autografts, bone substitutes such as xenografts and allografts, as well as platelet-rich plasma, have been proposed to enhance the outcomes of immediate implant placement and buccal gap augmentation procedures, with varying advantages and limitations [12, 13].

Autogenous bone grafts are considered the gold standard because they possess superior osteogenic, osteoinductive, and osteoconductive properties, with no risk of graft rejection or adverse reactions. However, their use is limited due to donor-site morbidity and unpredictable resorption [14].

Traditionally, allografts are obtained from donors of the same species and are available in different forms, including freeze-dried bone, fresh or frozen bone, and demineralized freeze-dried bone. These materials act primarily as osteoconductive scaffolds and may also exhibit osteoinductive properties due to the presence of bone morphogenetic proteins [15].

Allografts promote bone healing by enhancing osteoblastic activity and increasing the availability of growth factors, which accelerate bone formation and improve implant stability. They offer several advantages, including reduced patient morbidity, good biocompatibility, and minimal risk of immune or inflammatory reactions [16]. When used as a grafting material in the peri-implant gap, allografts can improve osseointegration, reduce marginal bone loss, enhance bone-to-implant contact, prevent soft tissue recession, and modulate the hard tissue healing process [17].

Because platelet concentrates can provide large amounts of leukocytes, autologous platelets, and growth factors that support bone formation and tissue healing, they have been used in numerous aspects of regenerative medicine [18, 19]. They are readily available, cost-effective, and rich in biological mediators that enhance tissue regeneration [20].

After centrifugation, platelet-rich plasma layers can be separated and used as a source of growth factors to enhance wound healing at surgical sites [21, 22].Platelet concentrates are now recognized as effective biological mediators that promote angiogenesis, accelerate revascularization, recruit various cell types, including stem cells, and stimulate cellular proliferation [23].

Growth factors, such as platelet-derived growth factor (PDGF) and bone morphogenic proteins, have been shown in numerous studies to promote bone formation in defective areas [24, 25]. PRF has demonstrated osteoinductive properties and the ability to accelerate bone healing in peri-implant defects [26].

However, the rapid resorption rate of PRF has historically limited its use as a barrier membrane, as it may not effectively prevent soft tissue infiltration over extended periods [27].

Recent advancements have shown that the resorption time of PRF can be extended through heat treatment of platelet-poor plasma, resulting in the formation of albumin gel. This technique, known as extended PRF (e-PRF), enhances the structural stability and prolongs the functional duration of the material [28].

In guided bone regeneration procedures, where a barrier function is required to prevent soft tissue invasion, e-PRF membranes can be used as an alternative to conventional collagen membranes [29].

Like plug materials or PTFE/collagen barriers, e-PRF membranes have been used for the management of extraction sockets following tooth removal in several clinical studies. This provides clinicians with a fully autologous biomaterial with prolonged resorption properties [30].

The objective of this study was to evaluate the effectiveness of allograft and extended platelet-rich fibrin (e-PRF) as space-filling materials during immediate dental implant placement.

## Patients and methods

### Patient selection

Twenty-eight patients requiring immediate dental implants to replace non-restorable maxillary anterior, first premolar, and second premolar teeth (esthetic zone) were selected from the outpatient clinic of the Oral and Maxillofacial Surgery Department, Faculty of Dentistry, Mansoura University.

Prior to the proposed surgery, written informed consent was obtained from all patients. All patients were informed about the benefits, risks, complications, and follow-up periods. All patients had the right to withdraw from the study at any time. The Institutional Review Board (IRB) of the Faculty of Dentistry, Mansoura University, Mansoura, Egypt, approved the current study in compliance with the seventh revision of the Helsinki Declaration in 2013 under protocol number (A0303024OS) on 12/03/2024. The study was following CONSORT guidelines for clinical trials. The study was listed on www.Clinicaltrials. gov with registration number (NCT07445776) on 03/02/2026.

### Criteria for patient selection

**Table:** 

Inclusion criteria	Exclusion Criteria
1. Patients with teeth that are badly destructed due to trauma or caries.	1. Individuals with systemic conditions that render dental implant placement unsuitable.
2. Patients aged between 18 and 50 years.	2. Patients who exhibit parafunctional habits (clenching and bruxism).
3. Patients who agree to complete the study follow-up period.	3. Alcoholics and heavy smokers (more than 20 cigarettes per day). [31]
4. Patients who maintain proper dental hygiene.	4. Patients with local infection or lesions at the proposed implant site.
5. Sufficient inter-arch space for subsequent restorations and implant abutments.	5. Individuals with untreated periodontal disease or poor oral hygiene.
6. No gender bias in patient selection. 7. Intact buccal bone 8. Jumping Gap dimension more than 2 mm [32–34].	6. Patients taking medications such as immunosuppressive drugs or bisphosphonates that may interfere with the repair of bones. 7. Patients unwilling to cooperate.

### Sample size calculation

The mean implant stability between the groups under study, obtained from earlier research by Elsheikh et al., [35] was used to calculate the sample size. Using a 2-tailed test, α error = 0.05, power = 90.0%, and an effect size of 1.27, the G Power program version 3.1.9.4 was used to compute the sample size. The total calculated sample size for each group was 14.

### Study design

This prospective randomised clinical trial adhered to the CONSORT guidelines for conducting clinical studies (CONSORT Flowchart). Patients were split into two groups at random:

#### Group I

14 patients received immediate implants where allograft was placed in the peri-implant gap.

#### Group II

14 patients received immediate implants where e-PRF was placed in the peri-implant gap.

### Randomization

The randomization and group allocation were done by a senior resident of the department who was not participating in the study and had no knowledge of any pertinent treatment regimens. Using a computer-generated randomization list (SPSS v25.0), 28 participants were split into two identical groups, each with 14 implants. The groups were distributed as follows: Group 1 received allograft while, Group 2 received e-PRF surrounding the implant at the gap distance.

### Blinding

The operator was not involved in the allocation or assessment procedures. However, patients were unaware of their group allocation. The outcome assessor performed all evaluations during the follow-up period without knowledge of the treatment provided. Similarly, statisticians were blinded to group allocation.

### Preoperative measures

Preoperative cone beam computed tomography (CBCT) was used to measure the buccolingual dimension, the angulation of the root and the amount of bone beyond the apex at the anticipated implant site using OnDemand 3D (Cybermed Co., Seoul, Korea). A prophylactic antibiotic regimen consisting of 500 mg amoxicillin (Emox, Egyptian Int. Pharmaceutical Industries Co., E.I.P.I.C.O., A.R.E.) every six hours was administered for two days prior to surgery [35] .Prior to the operation, instructions were given to the patients to rinse with chlorohexidine HCl (0.12%) (Hexitol, the Arab Drug Company, Cairo, A.R.E.) for one minute.

### Surgical procedures

After local anesthesia was administered (Mepecaine; Alexandria Pharmaceutical Co., Egypt), atraumatic tooth extraction was performed using periotomes to preserve the socket walls and to cut the periodontal ligament attachments. The tooth was then completely extracted using dental extraction forceps (Figs. 2 A, B, 3A and B). Drilling was done at 600–800 rpm in the proper direction. Sequential drilling with copious irrigation was carried out until the desired dimensions were reached, depending on the size of the implant. The implant type used was Conventional, two pieces, screw type titanium dental implant (Chaorum implant, Medimecca co., LTD, korea) with SLA (sand blasted, large grit acid etched) surface treatment, and the implant was inserted, leaving a gap distance between the buccal bone and the dental implant.

After implant placement, the buccal jumping gap was measured using periodontal probe to make sure that the distance from the implant surface and the buccal plate was more than 2 mm [32–34].

An Osstell Mentor device was utilized to verify implant stability using resonance frequency analysis (RFA). The SmartPeg (type 7) was attached to the dental implant (Osstell, Integration Diagnostics, Sävedalen, Sweden), and the cover screw was secured to the implant (Figs. 2 C and 3C).

**Fig. 1:**
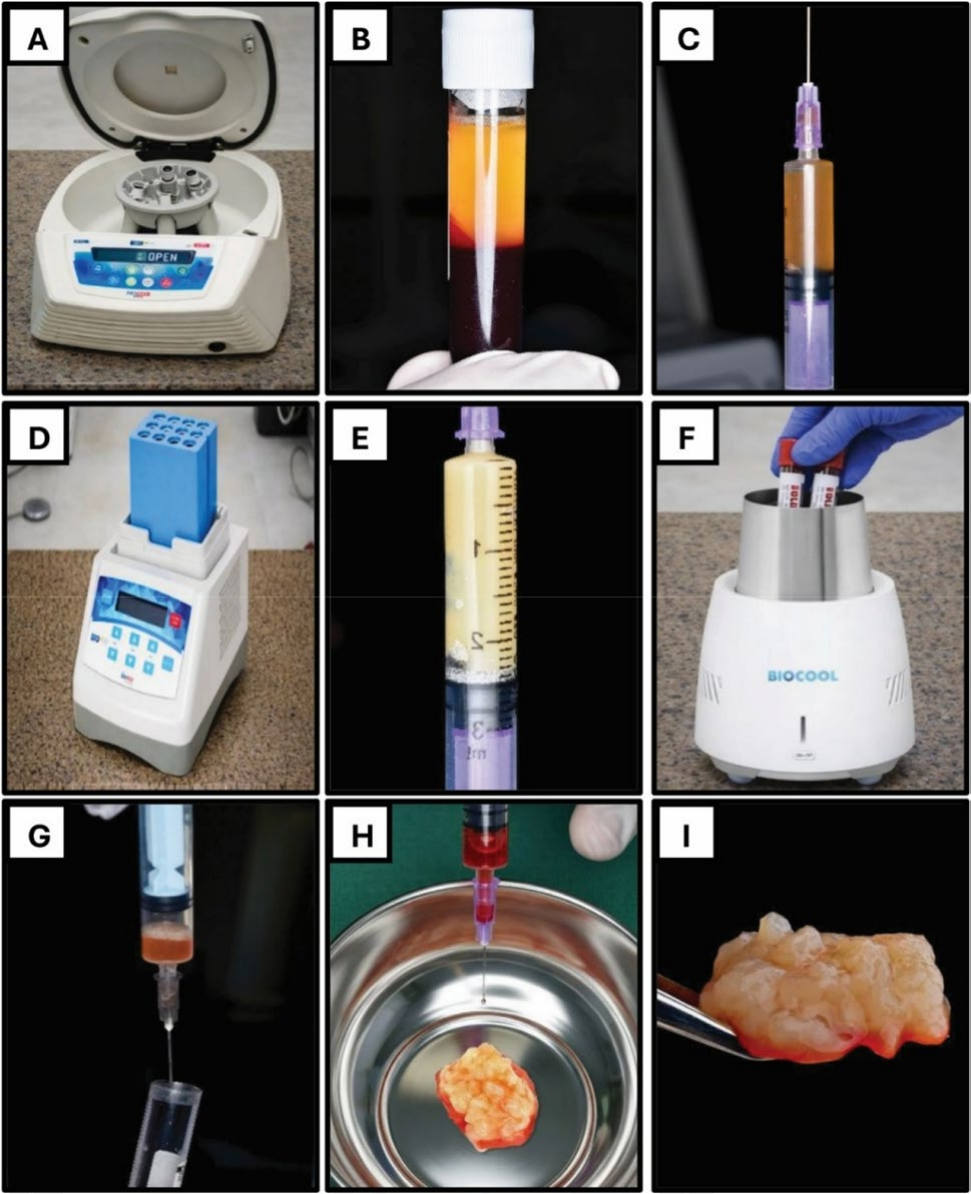
e-PRF preparation.** A** Whole blood was centrifuged at 2000 g for 8 min. **B** The upper layer (yellow layer) shows the liquid plasma layer. **C** The uppermost layer of platelet-poor plasma (PPP) was collected in a syringe. **D** The collected PPP was heated in a Bio-Heat medical device at 75°C for10 min and (**E**) The albumin gel was produced & was left to cool to room temperature for approximately 10 min. **F** liquid PRF was left in the Bio-Cool device to extend the clotting time. **G** The liquid platelet-rich layer (liquid-PRF), including the buffy coat layer with accumulated platelets and leukocytes, was collected in a separate syringe (**H**) the e-PRF was produced by mixing back albumin gel with liquid PRF. **I** a photograph showing the e-PRF membrane

**Fig. 2:**
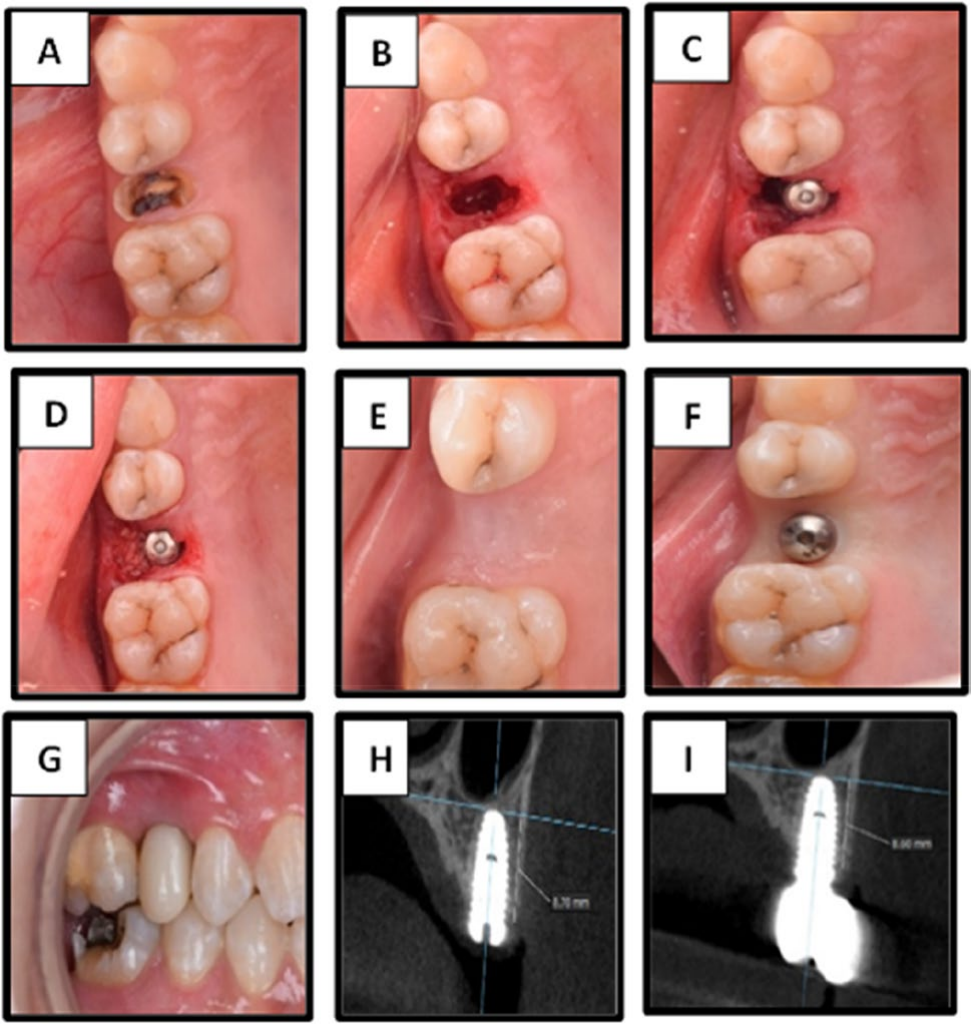
Allograft group. **A** A photograph showing badly decayed upper left second premolar. **B** Extraction socket after tooth removal without flap reflection. **C** Placement of Immediate implant. **D** Allograft after filling the gap around the dental implant. **E** The complete gingival healing after 1 month. **F** Healing abutment attached to implant (**G**) A photograph showing final restoration in place. **H** An immediate postoperative cross sectional CBCT image (**I**) A cross sectional CBCT image taken 12months postoperative

The buccal jumping gap: In Group I, the buccal jumping gap was packed using allograft (Regain-Oss, GARANTIE GmbH, Germany), which was hydrated and then placed in the gap around and over the dental implant (Fig. 2D), in Group II, the gap was packed using e-PRF (Fig. 2D). The clinical procedure for the preparation of e-PRF and albumin gel was performed as follows:

Peripheral blood was drawn using 9–10 mL tubes without any additives. Immediately after collection, the tubes were placed in a centrifuge (Bio-PRF, Horizontal Centrifuge, China, Relative Centrifugal Force (RCF-max): Approximately 2000 g for 8 min, Rotor Radius: Horizontal: 110 mm (max) / 88 mm (clot)) [30]. Following centrifugation, the blood components were separated into layers, including plasma and denatured red blood cells (Fig. 1A and B).

To prepare the albumin gel, 2–4 mL of platelet-poor plasma (PPP) was collected using a syringe and placed into a heating device for albumin denaturation (Fig. 1C and D). The PPP layer was converted into an albumin gel after 10 min at a temperature of 75 °C (Fig. 1E) [30]. To prevent rapid clotting, the concentrated PRF layer obtained from the buffy coat was placed into a cooling device (Fig. 1F).

After cooling, the albumin gel was transferred into a stainless-steel dish. The remaining 1–2 mL of concentrated liquid PRF (C-PRF) was then collected (Fig. 1G). The liquid PRF was mixed with the albumin gel in the stainless-steel dish and allowed to set for 15 min (Fig. 1H). The e-PRF membrane was then ready for use (Fig. 1I).

**Fig. 3:**
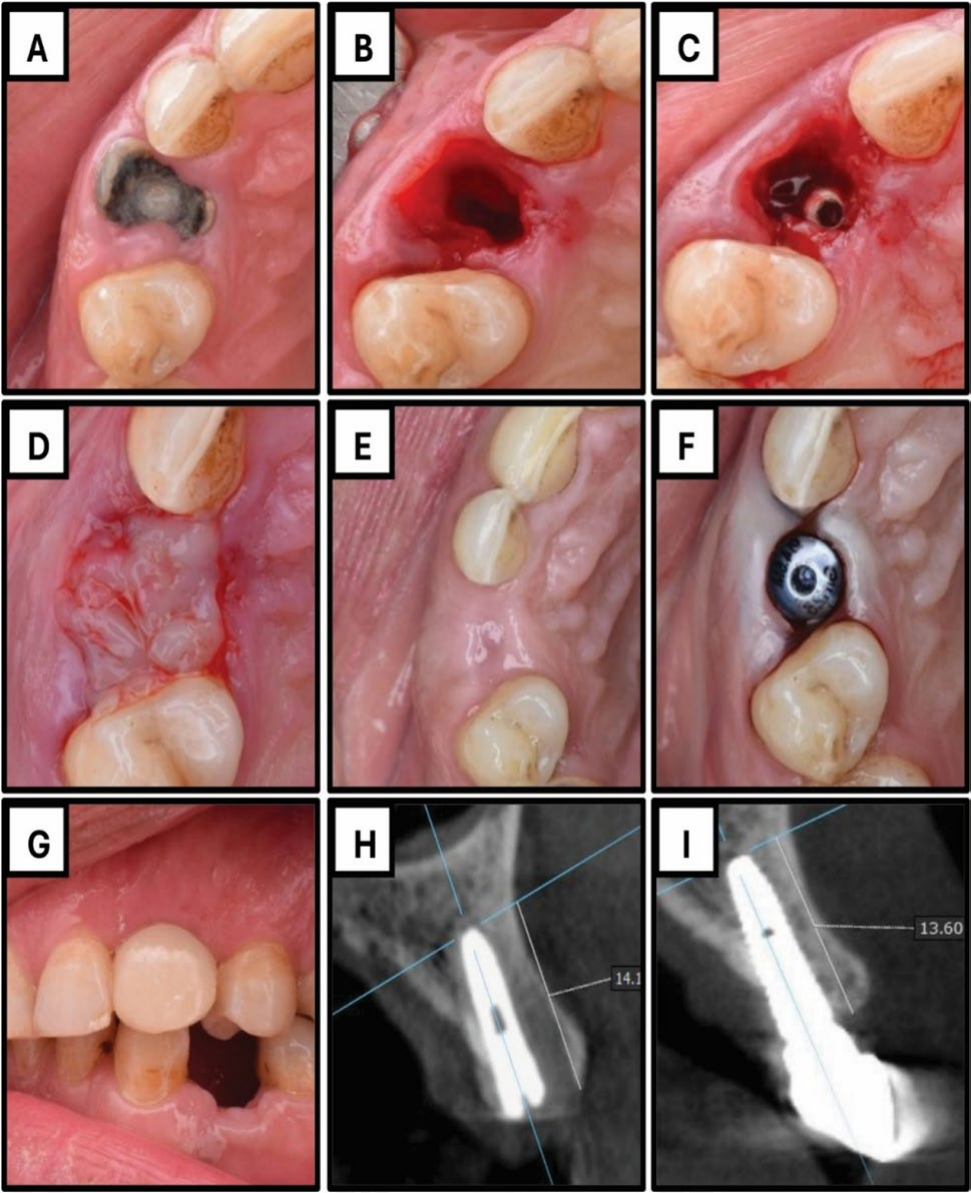
e-PRF group. **A** A photograph showing badly decayed upper left canine. **B** Extraction socket after tooth removal without flap reflection.; (**C**) Placement of Immediate implant. **D** e-PRF after filling the gap around the dental implant. **E** The complete gingival healing after 1 month. **F** Healing abutment attached to implant (**G**) A photograph showing final restoration in place. **H** An immediate postoperative cross sectional CBCT image. **I** A cross sectional CBCT image taken 12 months postoperative

The prepared e-PRF membrane was applied to the gap around the dental implant (Fig. 3D). Suturing was performed using (Proline suture 4/0, Ghatwry company, Egypt). Sutures were removed one week postoperatively. Final screw-retained zirconia crown restoration was applied after 6 months.

### Postoperative care and instructions

The patient was advised to use cold packs in the 1st day. Warm saline mouthwash was recommended starting from the day following surgery.

For seven days following surgery, all patients were given 500 mg of amoxicillin (Emox, Egyptian International Pharmaceutical Industries Co., E.I.P.I.C.O., A.R.E.) orally every six hours. Diclofenac potassium 50 mg tablets (Oflam, Mepha Pharma Egypt S.A.E.) were administered as a non-steroidal anti-inflammatory analgesic. Patients were advised to avoid biting on solid food and to maintain proper oral hygiene using chlorohexidine HCl (0.12%) (Hexitol, the Arab Drug Company, Cairo, A.R.E.). After one week, sutures were removed, and gingival healing was inspected after one month (Figs. 2E and 3E).

### Second stage surgery

A second-stage surgery was performed six months later. The cover screw was removed and replaced with a healing abutment for 15 days (Figs. 2 F and 3F).

### Prosthetic rehabilitation

A digital scan was performed to fabricate screw-retained zirconia crowns two weeks following soft tissue healing. The screw-retained zirconia crown was then placed after verifying marginal adaptation and occlusal contacts (Figs. 2G and 3G).

### Evaluation

Patients were evaluated at baseline (immediately), 6 months, and 12 months postoperatively.

#### A. Clinical Evaluation

### 1ry outcome

#### Implant stability

Implant stability was evaluated at the time of implant placement, as well as at 6 and 12 months postoperatively. A transducer was attached to the implant using a screw, and resonance frequency analysis (RFA) was recorded using the (Osstell Mentor device). Implant stability quotient (ISQ) values were obtained by averaging two measurements taken in two perpendicular directions (buccolingual and mesiodistal).

#### B. Radiographic evaluation

Radiographic evaluation was performed immediately after surgery (Figs. 2 H and 3H) and at 6- and 12-months (Figs. 2I and 3I) using cone beam computed tomography (CBCT).

The same CBCT machine (Morita CBCT; J. Morita Mfg. Corp., Kyoto, Japan) was used for all scans with standardized parameters (60–90 kVp and 1–10 mA). Image reconstruction and analysis were performed using OnDemand software.

### 2ry outcomes

#### Marginal bone loss (MBL)

Marginal bone loss was assessed vertically by calculating the average of three points: the most mesial point, the most distal point, and the center of the implant in cross-sectional views. The implant was utilized as a reference for the measurement of marginal bone loss (MBL) from the cross-sectional view by adjusting panoramic long axis in its center and bisecting it (showing the buccolingual dimensions).

**Fig. 4:**
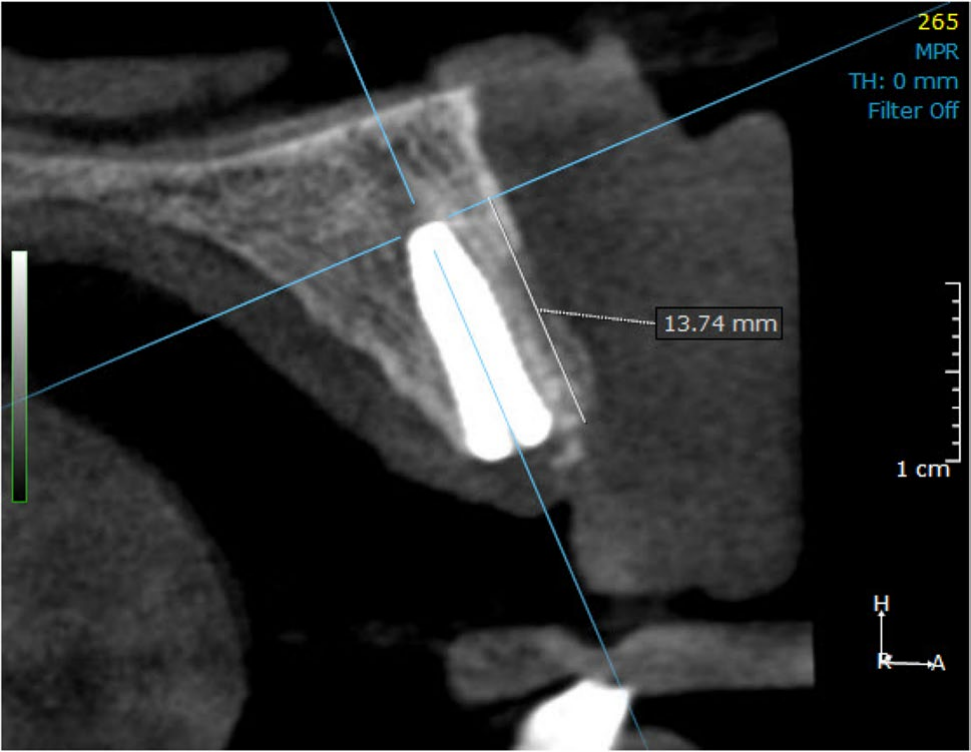
Measurement of MBL. A cross sectional CBCT image showing measurement of MBL represented by white line parallel to the implant, and its height was measured in millimeters at the crest of the buccal plate of bone and ending at the apical level of the implant. The implant was utilized as a reference for the measurement of MBL from the cross-sectional view by adjusting panoramic long axis in its center and bisecting it (showing the buccolingual dimensions) represented by blue lines

A line was drawn directly parallel to the implant, and its height was measured in millimeters at the crest of the buccal plate of bone and ending at the apical level of the implant. (Fig. 4). Measurements were taken immediately after implant placement and after 6 and 12 months. Changes in bone levels were calculated by subtracting follow-up values from baseline measurements.

#### Buccal bone thickness (BBT)

The implant was utilized as a reference for the measurement of Buccal bone thickness (BBT) from the cross-sectional view by adjusting panoramic long axis in its center and bisecting it (showing the buccolingual dimensions).

**Fig. 5:**
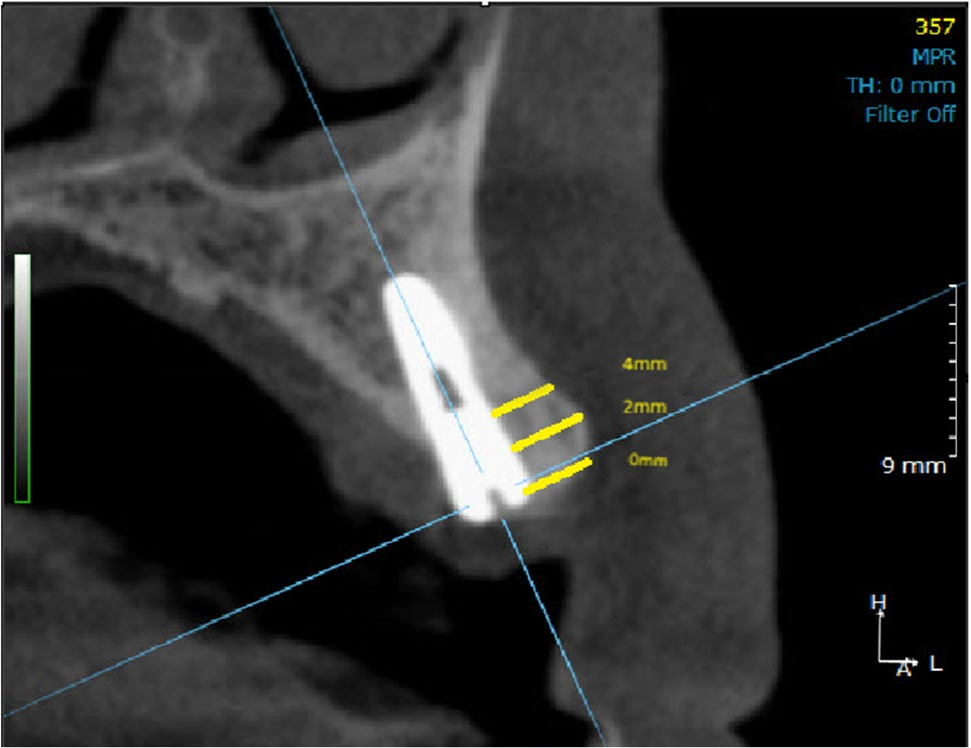
Measurement of BBT. A cross sectional CBCT image showing measurement of BBT, the implant was utilized as a reference for the measurement of BBT from the cross-sectional view by adjusting panoramic long axis in its center and bisecting it (showing the buccolingual dimensions) represented by blue lines. BBT was measured horizontally from the implant surface to the outer buccal bone plate at three different levels vertically represented by yellow lines 0 mm from implant crest, 2 mm below implant crest, and 4 mm below implant crest immediately after surgery

Buccal bone thickness was measured horizontally from the implant surface to the outer buccal bone plate at three different levels vertically represented by yellow lines 0 mm from implant crest, 2 mm below implant crest, and 4 mm below implant crest immediately after surgery. (Fig. 5). These measurements were considered baseline values. The same measurements were repeated after 6months and after one year, and the differences were calculated to determine horizontal bone loss.

### Statistical analysis

Statistical analysis was performed using SPSS software (version 26; SPSS Inc., Chicago, IL, USA). Qualitative data were expressed as numbers and percentages. Quantitative data were tested for normality using the Shapiro–Wilk test (appropriate for *n* < 50) and were expressed as mean ± standard deviation for normally distributed data, or median (minimum–maximum) for non-normally distributed data. The Mann–Whitney U test was used to compare two independent groups with non-normally distributed data, while the student’s t-test was used for normally distributed independent data. The Wilcoxon signed-rank test and paired t-test were used for intragroup comparisons.

To evaluate the clinical magnitude of findings, effect sizes were calculated (Cohen’s d for parametric tests; rank-biserial correlation, r, for non-parametric tests), and 95% Confidence Intervals (CIs) were computed for intergroup comparisons. To strictly control for Type I error inflation during multiple intragroup comparisons across the three time points (Immediate, 6 months, 12 months), the Holm-Bonferroni step-down procedure was applied. A p-value ≤ 0.05 was considered statistically significant prior to multiple comparison adjustments.

Parametric test was used for normally distributed data (used for measurement of implant stability and marginal bone loss).

Non-parametric test was used for non-normally distributed data (used for measurement of buccal bone thickness).

## Results

A total of 28 patients with non-restorable maxillary teeth in the esthetic zone (anterior and premolars) were treated with immediate dental implants. The patients were between 18 and 50 years of age (Table 1). All implants achieved adequate osseointegration throughout the follow-up period.

**Table 1:** Demographic data (Sex and Age)

Group Demographic data		Control group allograft n=14	Study group extended PRF n=14	
Gender	Female	10 (71.4%)	12(85.7%)	ꭓ2=0.848P=0.356
	Male	4 (28.6%)	2 (14.3%)	
Age	Mean ± SD	30.85±3.65	32.56±4.58	T=1.09P=0.284
	Range	25-45	28-48	

t: student t test ꭓ2: Chi-Square test, P<0.05 is statistically significant

Table (1): showing intergroup comparison regarding demographic data, shows no statistically significant difference between control and study group regarding gender and age

### Baseline comparability

At the immediate postoperative baseline, there were no statistically significant differences between the allograft and e-PRF groups regarding implant stability (*p* = 0.421), marginal bone loss (*p* = 0.517), or buccal bone thickness at 0 mm (*p* = 0.654), 2 mm (*p* = 0.338), and 4 mm (*p* = 0.085). This confirms the two groups were clinically comparable prior to the healing phase.

A. Clinical Evaluation

### 1. Implant stability

Implant stability did not differ statistically significantly between the control and study groups at baseline (*p* = 0.421), at 6 months (*p* = 0.878), and at 12 months (*p* = 0.391). When comparing implant stability within the same group at different time intervals, a statistically significant difference was observed (*p* = 0.001) (Table 2).

**Table 2:** Implant Stability (ISQ values)

Time Point	Control group (Allograft) n=14Mean ± SD	Study group (e-PRF) n=14Mean ± SD	t-value	p-value (95% CI)	Cohen's d
Immediate	49.85 ± 7.86	46.14 ± 8.80	0.833	0.421[-2.77, 10.19]	0.445
6 months	65.42 ± 4.07	65.14 ± 2.61	0.156	0.878[-2.37, 2.93]	0.082
12 months	71.85 ± 3.23	73.28 ± 2.75	0.890	0.391[-3.76, 0.90]	0.477

Table (2): showing intergroup comparison regarding implant stability, shows no statistically significant difference between control and study group at baseline (p= 0.421), after 6 months (p=0.878) and after 12 months(p=0.391)

B. Radiographic Evaluation

### 1. Marginal bone loss (MBL)

There was no statistically significant difference between the two groups regarding buccal marginal bone loss immediately after implant placement, at 6 months, and at 12 months (*P* > 0.05). However, when comparing intragroup changes, a statistically significant difference was observed between baseline and 12 months (*P* < 0.05) (Table 3).

**Table 3:** Marginal bone loss (MBL)

Time Point	Control group (Allograft) n=14Mean ± SD	Study group (e-PRF) n=14Mean ± SD	t-value	p-value (95% CI)	Cohen's d
Immediate	14.73 ± 2.21	14.11 ± 1.02	0.668	0.517[-0.71, 1.95]	0.360
6 months	13.74 ± 2.31	13.61 ± 0.86	0.137	0.894[-1.22, 1.48]	0.075
12 months	12.91 ± 2.01	12.64 ± 0.87	0.323	0.752[-0.93, 1.47]	0.174

Table (3): showing intergroup comparison regarding MBL, shows no statistically significant difference between control and study group at immediate (p=0.517), after 6 months (p=0.894) and after 12 months(p=0.752)

### 2. Buccal bone thickness (BBT)

Regarding buccal bone thickness, there was no statistically significant difference between the control and study groups at different levels or follow-up periods (*P* > 0.05) (Tables 4 and 5, and 6).

**Table 4:** Changes in Buccal Bone Thickness at 0 mm (Median (min–max)):

Comparison	Control (*n* = 14)	Study (*n* = 14)	*p*-value	Effect size (*r*)
Immediate – 6 months	0.90 (0.31–2.40)	1.12 (0.19–1.81)	0.654	0.085
Immediate – 12 months	1.06 (0.38–2.71)	1.88 (0.52–2.21)	0.406	0.157
6 months – 12 months	0.10 (-0.22–0.56)	0.50 (0.04–1.17)	0.199	0.242

Table (4): showing intergroup comparison regarding BBT at 0 mm, shows no statistically significant difference between control and study group values at Immediate -6 months (p=0.654), Immediate -12 months (p=0.406) and 6months - 12 months (p=0.199)

**Table 5:** Changes in Buccal Bone Thickness at 2 mm (Median (min–max)):

Comparison	Control (*n* = 14)	Study (*n* = 14)	*p*-value	Effect size (*r*)
Immediate – 6 months	0.77 (-0.18–1.78)	1.38 (-0.05–2.06)	0.338	0.181
Immediate – 12 months	1.19 (-0.01–1.46)	1.55 (-0.01–2.68)	0.124	0.291
6 months – 12 months	0.17 (-0.32–0.52)	0.37 (0.00–1.10)	0.482	0.133

Table (5): showing intergroup comparison regarding BBT at 2 mm, shows no statistically significant difference between control and study group values at Immediate -6 months (p=0.338), Immediate -12 months (p=0.124) and 6 months - 12 months (p=0.482)

**Table 6:** Changes in Buccal Bone Thickness at 4 mm (Median (min–max)):

Comparison	Control (*n* = 14)	Study (*n* = 14)	*p*-value	Effect size (*r*)
Immediate – 6 months	0.51 (0.31–1.16)	0.81 (-0.03–1.39)	0.085	0.325
Immediate – 12 months	0.76 (0.50–1.23)	0.92 (0.04–1.89)	0.179	0.253
6 months – 12 months	0.15 (-0.02–0.41)	0.07 (-0.03–0.05)	0.609	0.097

Table (6): showing intergroup comparison regarding BBT at 4 mm, shows no statistically significant difference between control and study group values at Immediate -6 months (p=0.085), Immediate -12 months (p=0.179) and 6 months - 12 months (p=0.609)

## Discussion

The successful integration of immediate dental implants depends on the proper control of the jumping distance, also known as the peri-implant gap, which exists between the buccal bone plate and the implant surface following implant insertion [36]. In contemporary implant dentistry, this gap represents a significant clinical challenge [37] .If not properly managed, it may compromise long-term implant stability and osseointegration. Complications such as peri-implantitis, marginal bone loss, and implant failure may occur [36].

Buccal bone thickness, implant stability, marginal bone preservation, soft tissue outcomes, and esthetic results after immediate implant placement are all influenced by the type of grafting material used [38].

Allograft is an effective osteoconductive material that acts as a scaffold, guiding the migration of osteoblasts and osteoclasts, allowing new bone formation, filling the peri-implant gap, and supporting osseointegration [38] .

The limitations of traditional grafting materials justify the investigation of e-PRF. Unlike conventional PRF, which undergoes rapid biodegradation within approximately three weeks, e-PRF maintains its structural integrity for a longer period, allowing the sustained release of growth factors such as TGF-β, PDGF, and VEGF [39] .These growth factors promote angiogenesis, accelerate healing, stimulate osteoblastic differentiation, and enhance bone formation, leading to improved implant stability and soft tissue healing [39] .

In the present study, implant stability was assessed using RFA. No statistically significant differences were observed between the two groups at implant placement (*P* = 0.421), at 6 months (*P* = 0.878), or at 12 months (*P* = 0.391). Primary stability was achieved by engaging the palatal wall and extending approximately 2 mm beyond the apex of the extraction socket.

When comparing implant stability within the same group over time, a statistically significant increase was observed (*P* < 0.001), which can be attributed to the transition from primary to secondary stability and increased bone-to-implant contact.

The findings of the present study regarding implant stability are in accordance with those of the study conducted by Elsheikh H et al. [40], who compared the efficacy of PRF versus alloplastic bone graft around immediate implants and reported that PRF increased implant stability over time.

A study by Jalaluddin M et al. [41] stated that allograft improved implant stability at different time intervals. Another study by Diana C et al. [13] reported that PRF increased stability around immediate implants and played a role in their osseointegration, as PRF gradually releases growth factors that induce osseous regeneration.

During the first year following loading, a successful implant typically exhibits an average marginal bone loss of 1.5 mm, followed by less than 0.2 mm annually when assessing marginal bone loss (MBL) [42].

In the present study, there was no statistically significant difference between the allograft and e-PRF groups regarding marginal bone loss immediately after implant placement (*p* = 0.517), after 6 months (*p* = 0.894), and after 12 months (*p* = 0.752).

There was a statistically significant difference in the intragroup comparison, indicating the average bone loss that normally occurs during the first year after loading.

The results of the present study are in accordance with those of a study by Jalaluddin M et al. [41], who compared allograft versus xenograft and showed that allograft placement in the jumping gap resulted in a statistically significant difference in intragroup comparisons. However, when comparing allografts and xenografts between groups, no statistically significant difference was observed.

A study by Nasef M et al. [43], which used allograft bone gel around immediate implants, demonstrated an effective role in implant osseointegration compared to non-grafted sites.

A study by Purohit M et al. [44, 45], which assessed hard and soft tissue alterations in immediately placed implants with and without DFDBA both radiographically and clinically, reported that bone loss was lower in the allograft group at the 12-month interval. The test group showed less crestal bone resorption compared to the non-grafted control group.

According to another study by Azangookhiavi H et al. [46], there was no statistically significant difference between PRF and FDBA in terms of marginal bone loss and alveolar ridge preservation during implant loading.

In a different study by Barakat M et al. [47] compared the effectiveness of e-PRF as an innovative autologous material used as a stand-alone graft alternative in two-stage lateral sinus lift procedures. E-PRF demonstrated significant clinical bone gain without the use of traditional grafts, with radiographic bone density averaging 322.7 ± 36.4 Hounsfield units (HU) and a mean vertical bone gain of 5.07 ± 1.78 mm six months after surgery. It was considered an excellent alternative due to its autologous nature and low risk of complications.

Our findings disagree with those of a study conducted by Nasrabadi N et al. [48], which stated that grafting the jumping distance with allograft had no significant effect on marginal bone level, as the use of a bone substitute is not mandatory for jumping distances up to 4 mm in immediate implant surgery.

At 0, 2, and 4 mm from the implant crest, buccal bone thickness (BBT) was measured. It was calculated using CBCT as the distance between the implant surface at each level and the outer buccal aspect of the alveolar bone. At all levels and follow-up periods, there was no statistically significant difference between the allograft and e-PRF groups in our study.

A study by Purohit M et al. [44, 45], which assessed the soft and hard tissue changes in immediately placed implants with and without DFDBA both clinically and radiographically using CBCT, reported that at the 12-month interval, there was a statistically significant difference in BBT values between the grafted and non-grafted groups at the level of the alveolar crest. However, BBT values were not significantly different at 5 mm in the control group or at 10 mm from the crest in both groups.

A study by Estrin N et al. [49], which compared four e-PRF membrane configurations with a traditional collagen membrane for alveolar ridge preservation, reported no statistically significant differences in buccal and lingual height, buccal bone thickness, or horizontal ridge width. Another study by Estrin N et al. [50], assessing the efficacy and safety of e-PRF membranes in ridge preservation, reported that the average change in lingual and buccal height was − 0.94 ± 1.07 mm and − 1.25 ± 1.16 mm, respectively. The average baseline buccal bone thickness at 1, 3, and 5 mm apical to the crest was − 1.14 ± 0.81 mm, − 1.37 ± 0.90 mm, and − 1.64 ± 1.06 mm, respectively. These findings suggest that the use of e-PRF membranes instead of collagen membranes for ridge preservation is a reliable, safe, and effective treatment option.

Another study by Moualla Z et al. [51], which used allograft in conjunction with e-PRF, showed slow resorption sufficient to support bone formation and achieve favorable outcomes regarding buccal bone thickness and vertical bone loss.

A study by Alsabahi H [52]., comparing a synthetic collagen membrane with an e-PRF membrane during immediate dental implant placement in the maxillary esthetic zone, reported that e-PRF showed reduced preservation of buccal bone thickness. In terms of marginal bone loss, e-PRF showed a statistically significant improvement, which may be attributed to its continuous delivery of growth factors. VEGF promotes angiogenesis, IGF enhances the osteogenic response, and PDGF and TGF-β stimulate osteoblast and mesenchymal stem cell proliferation and differentiation, thereby improving bone formation and reducing marginal bone loss.

When comparing the two grafting materials, e-PRF can maintain its structural integrity for a longer period due to its denser and more stable fibrin network compared to conventional PRF. One of its unique characteristics is the prolonged release of growth factors, which enhances its regenerative potential and facilitates the recruitment of osteoblasts and other progenitor cells involved in bone formation. Although allograft provides a more rigid and slow-resorbing scaffold for bone remodeling, e-PRF offers a viscous, moldable gel. While e-PRF is mechanically weaker than allograft, its distinguishing feature is the extended release of growth factors over a prolonged period, which may compensate for its lower structural rigidity. Therefore, both materials may contribute to bridging the gap between the implant surface and the socket walls, resulting in sufficient bone formation. Consequently, within this small exploratory trial, no statistically significant intergroup differences were detected between e-PRF and allograft regarding implant stability, marginal bone loss, and buccal bone thickness.

### Limitations

The limitations of this study include the relatively small sample size (*n* = 14 per group) and the short follow-up period of 12 months. Furthermore, the absence of an ungrafted negative control group limits the ability to distinguish the effects of the grafting materials from those of natural socket healing. Additionally, because the study was primarily powered to assess implant stability, it had limited statistical power to detect small intergroup differences in the secondary outcomes, namely marginal bone loss and buccal bone thickness. Future studies with larger sample sizes, longer follow-up periods, and appropriately designed non-inferiority or equivalence trials are recommended to provide more robust and generalizable evidence.

## Conclusion

Within this small exploratory trial, no statistically significant intergroup differences were detected between e-PRF and allograft regarding implant stability, marginal bone loss, and buccal bone thickness. Although e-PRF showed favorable clinical and radiographic outcomes and may represent a promising autologous regenerative material, further adequately powered randomized controlled trials, including ungrafted control groups, are required before conclusions regarding equivalence, non-inferiority, or its use as a definitive stand-alone graft alternative can be established.

## Supplementary Information

Supplementary Material 1.

## Data Availability

All data generated or analyzed during this study are included in this published article [and its supplementary information files].

## Abbreviations

e-PRF: extended Platelet Rich Fibrin
IIP: Immediate Implant Placement
CBCT: Cone beam Computed tomography
HU: Hounsfield units
ISQ: Implant stability quotient
MBL: Marginal bone loss
BBT: Buccal Bone Thickness
